# A study on sustainable air travel behavior under the possible remedy of risk knowledge: A mediating perspective of risk perception during COVID-19

**DOI:** 10.3389/fpsyg.2022.874541

**Published:** 2022-09-02

**Authors:** Warangsiri Niemtu, Kaida Qin, Muhammad Toseef

**Affiliations:** Faculty of Management and Economics, Kunming University of Science and Technology, Kunming, China

**Keywords:** sustainable air-traveling behavior, physical risk, psychological risk, risk knowledge, service quality

## Abstract

The aviation industry is the center of gravity for tourism-dependent countries seeking to uplift their economic activities. The COVID-19 pandemic in the early part of 2020 threatened people and the air industry to the maximum extent. This paper investigated the sustainable air travel behavior of passengers under the risk knowledge path. The mediating role of risk perception, i.e., physical risk, psychological risk, and service quality, was also tested for the risk knowledge-air travel behavior association. We surveyed 339 travelers at six airports in Thailand from January to June 2021 to record their responses. We applied covariance–variance-based structural equation modeling (CB-SEM), and the study results revealed a direct effect of risk knowledge with an indirect impact *via* risk perception paths on air travel behavior. This paper highlights knowledge as a remedial response to the perceptual makeup of air services sustainability. The study has solid managerial implications for aviation management in the design of ideal pathways for retaining air services during the current public emergency of COVID-19.

## Introduction

During the COVID-19 pandemic, passenger's travel behavior shifted significantly. Aviation management calls for the perceptual positioning of passengers as a remedial tool for sustained travel demand (Song and Choi, 2021). The enduring damage continues to rise in tourism, transportation, catering, entertainment, and retail due to COVID-19. People perceive the current pandemic in terms of physical threat, employment loss, home-to-home disease transmission, suffering, and death (IATA International Air Transport Association, 2019; Zhu and Deng, 2020). In the initial 3-month phase, the aviation industry bore 70 to 95% decline in passenger demand with passenger traffic disruptions (Conway III et al., 2020; Spitzmuller et al., 2020). On the other hand, the aviation industry has experienced the decades of steady growth, even during the 9/11 terrorist attacks of 2001 and the global economic crisis of 2008. The aviation industry has maintained a consistent annual growth rate of up to 4.5% (Shepardson et al., 2020). Historically, H1N1 and severe acute respiratory syndrome (SARS) diseases have impacted air travel's domestic and global stature, which is also the current case of the public emergency during COVID-19. The outbreak of the new coronavirus at the start of 2020 isolated all of China, not only from people but also from industry (Whitely et al., 2020). COVID-19 exerted immense pressure on various industries in China and around the world as the outbreak spread.

**Figure 1:**
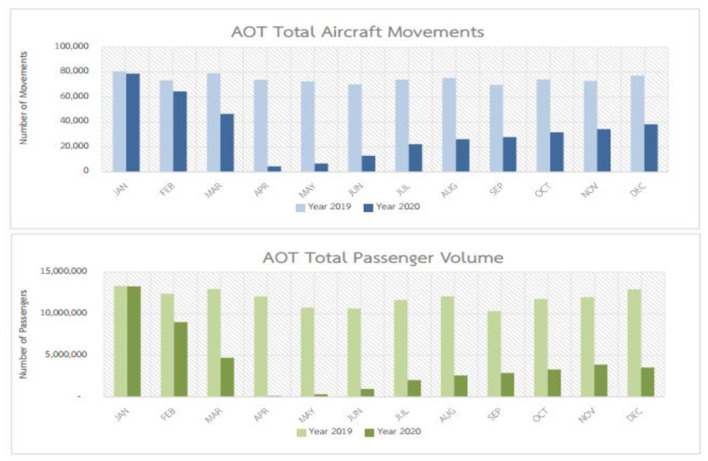
Comparative position of aircraft movement and passenger volume 2019–2020.

As a neighboring country, Thailand also came into contact with COVID-19 in January 2020. Thousands of travelers use Thai airports to return home, which gave rise to the virus in the country. The report of Adrienne et al. (2020) elaborated that outbreak of the pandemic dramatically declined air traffic in Thailand, and the passenger volume and aircraft movement decreased by 55.78 and 67.39%, respectively (refer to Figure 1).

Scholars such as Bellizzi et al. (2019) argue that risk perception is a pathway to judgmental uncertainty in the tourism market. Moreover, people's preferences and behaviors are influenced by their contact with public health emergencies in the consideration of perceived risk. The prior work of Airport of Thailand PLC. (2020) and Aircraft Traffic Report (2020) reported consumption behavior in tourism and identified the contribution of perceived risk in comparison with perceived value. Moreover, the pull–push theory of Li (2008) and Zhao (2012) argued that traveling needs and purposes stem from the primary motive of people's willingness to travel. The work of Roehl and Fesenmaier (1992) incorporates quality service as the only possible tool to fill people's travel needs by adapting to the precausal global health machinery. Moreover, they argue that quality of service captures consumption trust and behavioral intention with regard to service offers.

Few ongoing studies have highlighted air transportation as a path of virus spread, and countries across the world have implemented policies to halt air service (Mitchell and Vassos, 1998; Zhang et al., 2018; Shah et al., 2020). Thus, there is a need to investigate a realistic path model to manage the aviation industry during the postpandemic period in a tourism-sustained country. In this regard, working aviation-machinery executives are mapping passenger awareness as a viable path to overcome the existing challenges in commercial aviation and as a new and distinctive approach based on physical and psychological safety (Conway III et al., 2020). This paper is based on three novel points (Durande-Moreau and Usunier, 1999). First, travel knowledge is critical for behavioral preferences during the pandemic at the destination point in addition to the knowledge of pneumonia (Durande-Moreau and Usunier, 1999; Zhu and Deng, 2020), usefulness, timing, and facilities. Second, airline's perceptions of service quality hold the behavioral key to travel, willingness to use resources, and intention to fly during COVID-19. Third, the theory of knowledge-attitude-behavior (KAB) is chosen to establish a constructive path between risk knowledge and the behavioral intention of air travel *via* risk perception.

## Literature review

COVID-19 is a recent issue of concern for airlines to address and around which to establish policy for the sustainability of air services management. Prior studies have been confined to foreseeing passengers' behavioral changes, risk knowledge, physical and social servicescapes, satisfaction in connection to sustainable airport image, and travel behavior (Conway III et al., 2020; Shah et al., 2020; Song and Choi, 2021). In association with COVID-19, aviation management must take precautions to stimulate and sustain air service as the only option for transportation management under the World Health Organization (WHO) guidelines. This article used a deductive method to examine air travel behavior by consolidating service quality and travel knowledge in a conceptual model.

Individual perception is the central factor of risk psychology, which focuses on humans' information processing capacity as they develop social awareness and responsiveness to risk.

### Behavioral intention

The study of Molinari et al. (2008) elaborated upon the conceptual position of behavioral intention as the potential future actions of individuals to forecast human behavior. Zeithaml et al. (1996) considered five dimensions of behavioral sustainability: willingness to pay, internal and external responses to problems, switch, and traveler loyalty. A study on consumer's behavior reported that customer retention and potential buying services reflect repurchase intention. Furthermore, customer's satisfaction consolidates the aviation industry's positivity, feedback, and reuse of services. In the context of the service sector, researchers have adopted tridimensional measures proposed by Park and Ryu (2019), that is, willingness to pay, intention to reuse airports for investigating behavioral intention (Cameron and James, 1987; Hanemann, 1991; Cronin Jr and Taylor, 1992), and subsequent hypotheses in the pandemic context. In this study, KAB theory was adopted to develop a risk knowledge-risk perception-behavioral intention association. The KAB model establishes a continuous process of acquiring knowledge, generating beliefs, and forming behavior (Zhao, 2012). Other models, such as the theory of planned behavior (TPB), emphasize subjective norms and sustainable behavior in connection with knowledge, attitudes, and behavior in the external knowledge domain of consumer behavior. The KAB model has wide application in prior education studies, public health, clinical medicine, and other social aspects. A partial mediation effect of attitude was found by Zhang et al. (2012), and a direct positive association of knowledge, attitude, and behavior was verified along with an indirect association of knowledge in a hypertension study by Zeng et al. (2017).

### Hypothesis development

The immediate consensus of the aviation industry is that a full recovery is required to allow for the development of optimistic guidelines for airlines (Fasanelli et al., 2020; Miller et al., 2020; Taylor et al., 2020; Unnikrishnan, 2020). Travel industry documents, crises, and cultural and functional risks are undermining sustainable traveler perception. Furthermore, it has been argued that social, physiological, psychological, time, satisfaction, capital, and security risks affect travel perceptions in the service sector over a sustained period of time (Harrison-Walker, 2001; Joffe, 2003; Floyd et al., 2004). With the COVID-19 pandemic, airlines have engaged directly with their customers, most often *via* email, to reassure passengers about the safety steps they are taking such as rigorous cleaning, disinfecting, and social distancing processes. Passengers who seek to enhance their lives through tourism develop travel habits characterized by a desire to seek adventure; however, such tourism habits are also associated with risk reduction attitudes that have an impact on tourism behaviors (Mo et al., 2002; Wang et al., 2019).

Risk perception can be as the subjective judgments of consumers regarding tourism, safety information, crisis events, and shortcomings related to travel (Moutinho, 1987; Mowen and Minor, 1988; Cater, 2006), meaning that the developments of knowledge proceed subjective norms and reach the behavioral destination of humans. As long as the people came in to contact with the situational awareness, they will shift toward the acceptance of personal decisions.

Risk perception has been reported to be associated with health risks, social behavior, psychological attitudes, satisfaction, risk knowledge, and behavioral intention (Zhu et al., 1988; Chai et al., 2011; Chen and Zhu, 2015; Guo et al., 2015; Liu, 2019). Studies have reported that the linkage of risk perception with service quality has a great impact on customers' judgments and evaluations of organizational services in terms of empathy, responsiveness, reliability, and tangibility (Zeithaml, 1988; Bateson and Hoffman, 2002). Second, the study hypothesized the relationship that explained that the information regarding the uncertainty cause perceptual build that is physical, psychological, and service concerns of the travelers in the presence of ground reality called COVID-19. People search for the physical and psychological damages along with service adjustments made by the airliners. As long as the knowledge contributes, the perceptual stability can be secured and the aviation industry moves back to the service retention.

The literature and theories reflect the inclination of perceptual risk toward intention development. Here, the behavioral intention stems from the human perception, i.e., how physical, psychological, and service domains are critical to mold the human behaviors.

**Figure 2:**
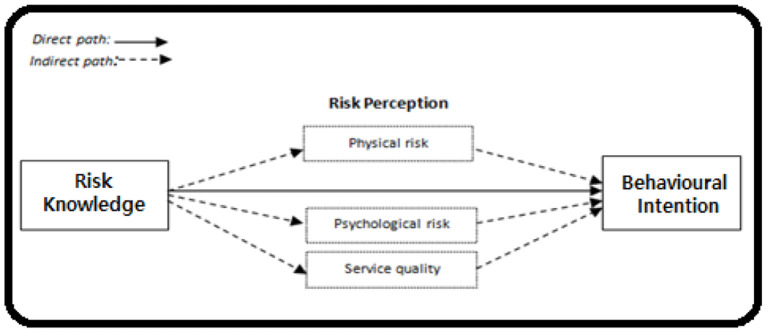
Conceptual model of the study.

Prior studies have identified a connection between service quality and behavioral intention (Cho and Hu, 2009; Alrubaiee and Alkaa'ida, 2011; Shah et al., 2020). The study supposition supported by the previous work explained the mediating path. The hypotheses explained that physical risk is causing the association of knowledge toward traveling behavior. The role of knowledge which the people of Thailand been guaranteed by the social and official sources to make decision steps to travel. Therefore, psychological risk is the central point of concern in the current emergency. The psychological ground connects behavioral intention by the sources of situational knowledge that is isolation or precautions to travel. Service quality is the only practical tool in all walks of life to continue the services for the aviation industry, which ask for the awareness, understanding required by the airlines to reach mental state of decisions to move. The conceptual framework of the study positioned variables highlights the path of reaching the behavioral intention of the air travel behavioral intention under post COVID-19 (refer to Figure 2).

### Risk knowledge and behavioral intention

The research of Zhao (2012) reported 3-D knowledge domains, general, social, and significant, generated from outside interaction, vast-access information, and specialized knowledge fields. Individual tension preferences connect risk based on the knowledge obtained by traversing the civic circle. Moreover, the study found that information promotes risk-taking behavior. Complete information has been found to cause people to act rationally when they are exposed to risk whereas they will follow risk avoidance under incomplete information (Zhang and Cheng, 2008). Knowledge sustainability is associated with passengers' intention to rejoin travel, which had been limited due to the restrictions associated with the COVID-19 pandemic. In terms of financial loss, the distinctive positioning of the risk mapped by Mitra et al. (1999) is directly associated with product sustainability. Additionally, problematic and damaging product or service characteristics impact the consumers' perception and increase their sense of psychological risk.

In contrast, consumer physical damage is caused by a poor-quality service course for physical risk. Social risk refers to probable negative comments received in the context of family, work relations, and decision-making. Additionally, travel behavior post-COVID-19 demands the sustained efforts of air travel management to synchronize service utility as a performance risk along with time consumption in decision-making.

The empirical findings in medical science are connected to the spread of risk knowledge to lower perception and guide experts' behavior in the direction of medical care (Daniali et al., 2015). Furthermore, perceptual risk is the commencement of subjective belief related to the adverse behavior of catching a disease. The cognitive components of individuals have a stronger association with knowledge obtained through extended work behavior during public emergencies (Glanz et al., 2008). Consequently, another study exposed opposite findings: poor precautionary practices among individuals were seen under knowledge and attitudes. That study further highlighted that work practices are continuously exposed to public risk. Under such conditions, the work context places demand on the individuals to manage uncertainty and obtain sustainable human movement to sustain civic circles.

**H1**. Risk knowledge significantly influences behavioral intention of air travel during the pandemic in Thailand.

### Risk knowledge and risk perception

According to a study in the travel domain, the risk of health, terror, and natural disasters necessarily requires knowledge, awareness, and experience to attain a traveling attitude. Similarly, in the context of international tourism, a wealth of risk knowledge leads to a less perceptual risk position and a reduction in human uncertainty or unfavorable outcomes with decisive positions (Dowling and Staelin, 1994; Lepp and Gibson, 2003). The empirical work of Johnson et al. (2008) revealed that an unawareness or zero or minimal risk stems from high-risk perception and structures adverse consumption decisions.

Travel industry documents, crises, and cultural and functional risks lift the constructs of sustainable traveler perception. Another study argued that social, physiological, psychological, time, satisfaction, capital, and security risks affect travel perceptions in the service sector over a sustained period (Stone and Grønhaug, 1993; Floyd and Kirby, 2001; Lou, 2004). Additionally, service risk also requires knowledge to tackle uncertainty by designing sustainable tourism policy. The study of Lepp and Gibson (2003) in the international travel circle found that rich knowledge about travel, food, and health will operationalize and control perceived risk. Chai et al. (2011) verified the sustained predictive power of risk knowledge for risk perception, and Wang and Xu (2020) studied the negative significance of risk knowledge and public perception along with interest involvement and information saturation. Prior work has explained that rational choices mitigate potential risks by adopting risk sustainability while working under unfavorable circumstances.

**H2**. Risk knowledge significantly influences the perceived physical risk of sustainable air travel during the pandemic in Thailand.
**H3**. Risk knowledge significantly influences the perceived psychological risk of sustainable air travel during the pandemic in Thailand.
**H4**. Risk knowledge significantly influences the perceived service quality of sustainable air travel during the pandemic in Thailand.

### Risk perception and behavioral intention

According to Mowen and Minor (1988), perceived risk is the likelihood of unfavorable outcomes. People with various personal qualities perceive varying dangers in the same mode of transportation (Hanemann, 1991) [20]. Medical experts believe that individuals with underlying medical problems, such as heart disease, obesity, asthma, and diabetes, may have an elevated risk of sickness and death from COVID-19 (Nania, 2020). Those who cannot maintain a sufficient level of health care are unlikely to be ready to subject themselves to the enduring danger of incurring more medical expenses. Families with children or vulnerable members may be less inclined to risk harming a family member due to the new coronavirus (Lamb et al., 2020).

According to a survey, people's willingness to travel by air will drastically decrease, and the sustainability of travel behavior could be a point of concern for airlines (IATA International Air Transport Association, 2020). As a result of the significant decrease in passenger loads post-COVID-19, airlines are engaging directly with their customers, most often *via* email, to reassure passengers about the safety steps they are taking such as rigorous cleaning, disinfecting, and social distancing processes (Shepardson et al., 2020).

Another study found that evaluating the quality of services that a business offers determines customer's trust in that company and sustains consumers' behavioral intention. An empirical study of Zeithaml et al. (1996) established SERVQUAL, which assesses service quality along five dimensions: tangibles, dependability, responsiveness, assurance, and empathy. This method is intended to assess customer's satisfaction by measuring consumer's expectations and perceptions. The study utilized SERVQUAL to assess the service quality of airlines from the perspective of foreign passengers. Passengers regard comfortable seats and cleanliness to be vital services that any aircraft company can provide to obtain sustainable competitive advantages (Liou and Smith, 2007).

Furthermore, passengers place a premium on “safety-related services” in the aviation sector. Behavioral intentions are viewed as a consequence of service quality, influencing customer's behavior and, ultimately, the firm's financial situation. The work of Hua et al. (2010) demonstrated a direct negative link among safety concerns, geographical damage, casualties and damage to facilities and equipment, psychological taboo, ethical problems, financial concerns, and tourist intention. Yingzhi et al. (2015) investigated the adverse effects of social, political, and cultural risk on Japanese tourist intention. The majority of studies have concentrated on the impact of service quality on tourist intention. Transportation convenience, tourist safety, lodging convenience, the level of tourism information, travel agency services, leisure time, and conforming psychology all have been positively linked with tourism intention (Jia, 2018; Gong and Du, 2019). The connectivity of COVID-19 influences the sustainable behavior of passengers, which leads us to propose the following hypotheses:

**H5**. Perceived physical risk significantly influences sustainable air travel behavioral intention during the pandemic in Thailand.
**H6**. Perceived psychological risk significantly influences sustainable air travel behavioral intention during the pandemic in Thailand.
**H7**. Perceived service quality significantly influences sustainable air travel behavioral intention during the pandemic in Thailand.

### Mediating effect of risk perception on risk knowledge-behavioral intention

One work Bae and Chang (2021) positioned traveling as a universal human need of modern individuals. The health belief model (HBM) reported that the congruence of copious risk perception lifted an individual's health-protective sustainable behaviors (Rosenstock, 1974). Prior studies have widely discussed health-seeking and health-protective behaviors and prolonged travel decisions in the context of health emergencies reported historically, including SARS and avian flu (Floyd and Pennington-Gray, 2004) and non-pharmaceutical intercession for disease (Yu et al., 2012). A Korean study on travel intention during the pandemic located citizens' travel trust following isolation by capturing the subjects' travel knowledge path curtailing perceived risk (Choi et al., 2021). The binary dimensional concept of cognitive and affective perceived risk also affirms the susceptibility, severity, and anxiety levels related to an individual's exposure to risk (Sjöberg, 1998, p. 52).

Furthermore, there is strong evidence that service quality has either a direct or indirect effect on customer's sustainability behavioral intentions, which is mediated through customer's satisfaction (Zeithaml et al., 1996). A survey of 457 medical students concluded that health safety, for example, in the transmission of hepatitis C, is available to individuals who seek knowledge to control the perceptual positioning of hepatitis C virus (HCV) and capture behavioral intention toward medical care. In the marketing domain, the study of MacInnis et al. (1991) mentions that an abundance of knowledge structure led to a decline in risk perception and the arousal of sustainable motives toward purchasing intention. Moreover, behavioral intention is directly associated with product uncertainty knowledge value control (Singh and Smith, 2005; Teo et al., 2009). The empirical work of Van Fan et al. (2021) in the Chinese service sector studied the factor of information disclosure intention and concluded that perceived risk is a contributor to behavioral intention through transparent information processing.

**H8**. Perceived physical risk significantly mediates risk knowledge and the behavioral intention of sustainable air travel during the pandemic in Thailand.
**H9**. Perceived psychological risk significantly mediates risk knowledge and the behavioral intention of sustainable air travel during the pandemic in Thailand.
**H10**. Perceived service quality significantly mediates risk knowledge and the behavioral intention of sustainable air travel during the pandemic in Thailand.

## Methods

### Population and sampling

The top six Thai airports with the most passengers in the first quarter of 2021 are Bangkok Don Mueang, Bangkok Suvarnabhumi, Chiang Mai, Phuket, Hat Yai, and Nakhon Si Thammarat, which are taken as the population (*N* = 5.05 million) of the study (refer to Table 1). Multistage sampling is used, starting with non-probability using Yamane (1967) formula, and a sample (*n* = 399) is drawn from the population. The travelers traveling through the airports came into contact with the study by the online link provided at the airports (Figure 4).

Yamane's formula = N/1+N(e)^2^
Total population: *N* = 5,050,000
Error: e = 0.05
Sample: *n* = 399

**Figure 3:**
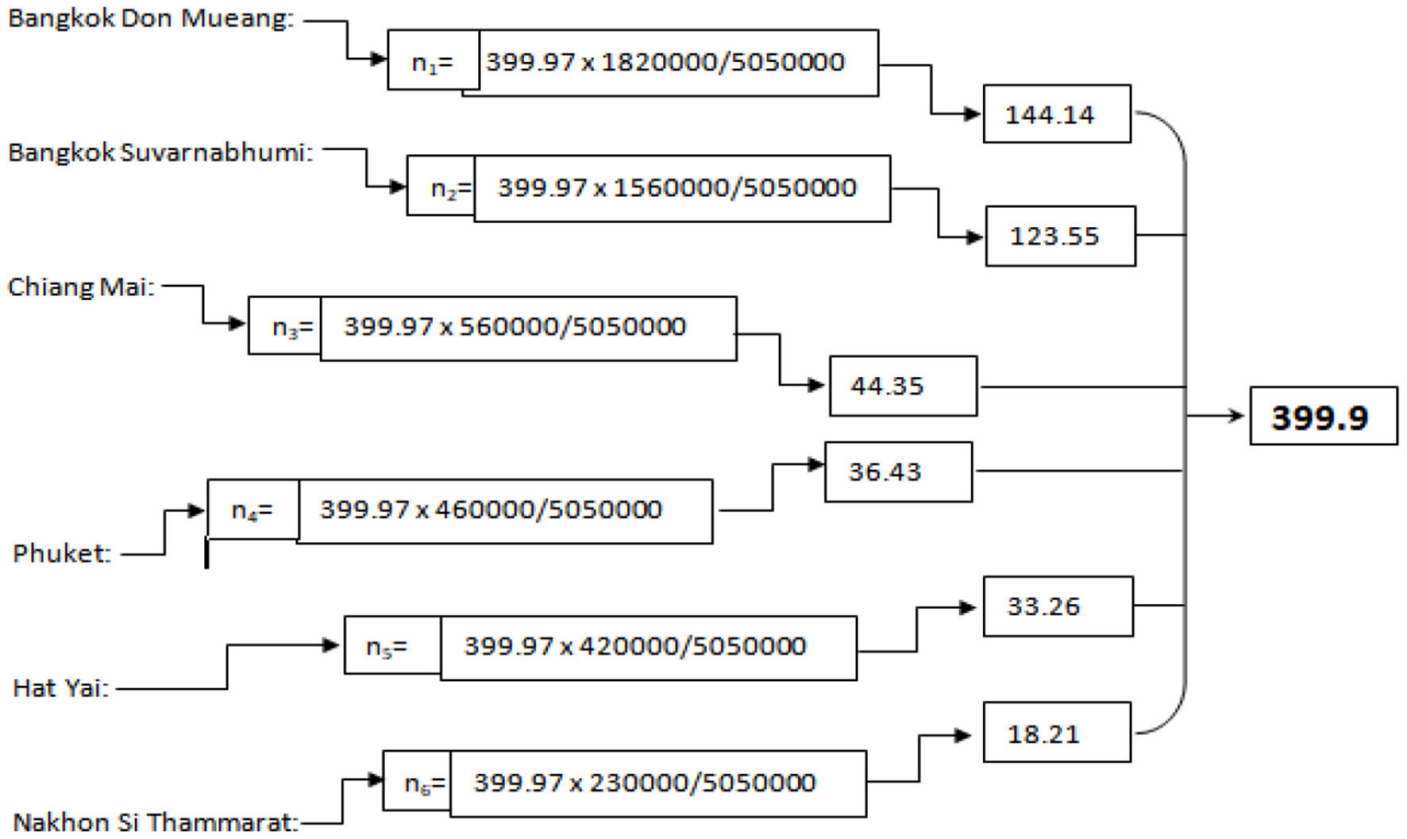
Sample estimation.

Then, the stratified sampling formula is used to obtain a proportionate sample from each study strata. The sample of each strata is as follows: n_S_= targeted population x sampling population/total population (refer to Figure 3).

**Table 1:** Statistics of air service users in the first quarter of 2021.

**S. No**	**Thailand airports**	**Number of passengers**	**% Decrease in passengers**
1	Bangkok Don Mueang	1.82	−76.2%
2	Bangkok Suvarnabhumi	1.56	−87.0%
3	Chiang Mai	0.56	−74.1%
4	Phuket	0.46	−88.0%
5	Hat Yai	0.42	−46.5%
6	Nakhon Si Thammarat	0.24	−25.8%
7	Chiang Rai	0.23	−61.7%
8	Udon Thani	0.22	−59.2%
9	Ubon Ratchathani	0.19	−50.6%
10	Khon Kaen	0.18	−55.0%
11	Surat Thani	0.16	−63.0%
12	Krabi	0.14	−82.9%
13	Trang	0.08	−49.6%
14	Ko Samui	0.07	−86.7%
15	Phitsanulok	0.06	−57.2%

Airports of Thailand Public Company Limited, Department of Airports, U-Tapao Airport Authority and Bangkok Airways Public Company Limited: Analysis by the Aviation Economy Division.

**Figure 4:**
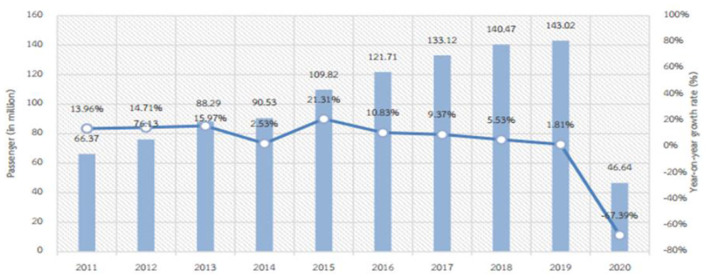
Passengers (in millions) and % change 2011–2020.

Participant willingness and privacy are assured, along with the data separation to handle the common method bias proposed in the study of Podsakoff et al. (2012).

The statistics for passengers at the top 15 airports in Thailand indicate a substantial decline in the first quarter of 2021 compared to the previous year. Bangkok Don Mueang airport had 1.82 million travelers, a 76.2% decrease; Bangkok Suvarnabhumi had 1.56 million, an 87.0% decrease; Chiang Mai had 0.56 million, a 74.1% decrease; Phuket had 0.46 million, an 88.0% decrease; and the fifth airport Chiang Rai had 0.23 million, a 61.7% decrease. The statistics for these six airports are shown in Table 1.

### Instrument

This paper emphasizes an adopted instrument for measuring five variables using a five-point Likert scale. The variable risk knowledge is measured using a 10–item scale (Zhao, 2012; Liu, 2019; Xu et al., 2019). The constructs, which include 3 items of physical risk and 4 items of psychological risk from Han (2005) and 4 items of service quality at airports (Hutchinson et al., 2009; Ryu et al., 2012), represent risk perception. Moreover, a 13-item scale was used to measure the behavioral intention of Zhao et al. (2016); Xu et al. (2019); Rice et al. (2020) air travel considering the Thai aviation industry. The questionnaire contained age, gender, education, and travel frequency to obtain the demographic profile following the variable assessment. The items were adopted from a previously validated psychometric scale, and earlier researchers have validated the lack of redundant items associated with the scale used in this study.

The graph displays the progression of air services in Thailand over 10 years. In 2011, 66.37 million people used air services in Thailand (13.96), increasing the number of passengers. However, in 2019–2020, passengers decreased from 143.02 to 46.46 million, and the decline rate was 1.81 to −67.38 due to COVID-19 (Aircraft Traffic Report, 2020).

### Data collection and analysis

Data collection was performed for 6 months, starting in January 2021 and covering the first quarter of that year. The researcher adopted an online survey using Google Form as a practical tool to approach passengers in the presence of COVID-19 with their compulsory agreement option to be a part of this study under research ethics. The simultaneous connectivity of the constructs for path analysis is a central point of structured equation modeling (SEM); in this study, analysis of moment structures (AMOS) is used. Covariance-based SEM holds a theory testing edge in a single complex model (Chin et al., 2003).

## Findings

Table 2, which provides a demographic representation of the participants, indicates 163 men (40.9%) and 236 women (59.1%); 67 individuals aged 21–30 years (19.0%), 31–40 years (37.6%), 41–50 years (24.8%), and 74 of them above 50 years (18.5%); 34 individuals with higher secondary education (8.5%), 216 with a Bachelor's degree (54.1%), and 149 with a Master's degree (37.3%). Furthermore, the participants' travel frequency was also categorized as 133 one time per year (33.3%), 73 two times per year (18.3%), and 193 more than two times per year (48.4%).

**Table 2:** Demographic categorization.

**Item**	**Options**	**Sample**
Gender	Male	163 (40.9%)
	Female	236 (59.1%)
Age	21–30 years	76 (19.0%)
	31–40 years	150 (37.6%)
	41–50 years	99 (24.8%)
	Above 50 years	74 (18.5%)
Education	Higher secondary	34 (8.5%)
	Bachelors	216 (54.1%)
	Masters	149 (37.3%)
Travel frequency	One time per year	133 (33.3%)
	Two times per year	73 (18.3%)
	More than two times per year	193 (48.4%)

### Confirmatory factor analysis

This section investigates the model of risk knowledge pathways to the behavioral intention of air travel risk perception during COVID-19 using confirmatory factor analysis (CFA). The objective of this CFA was to verify the adequacy of items for the factors and the number of dimensions underlying the construct in this empirical model (Bollen, 1989). The findings indicate that the confirmatory factor analysis (CFA) of the model of risk knowledge pathways to the behavioral intention of air travel risk perception during the COVID-19 model identifies five latent variables with a total of 30 observable variables including psychological risk perception (PSR), physical risk perception (PR), service quality (SQ), risk knowledge (RK), and behavioral intention (BI) (Table 6).

**Figure 5:**
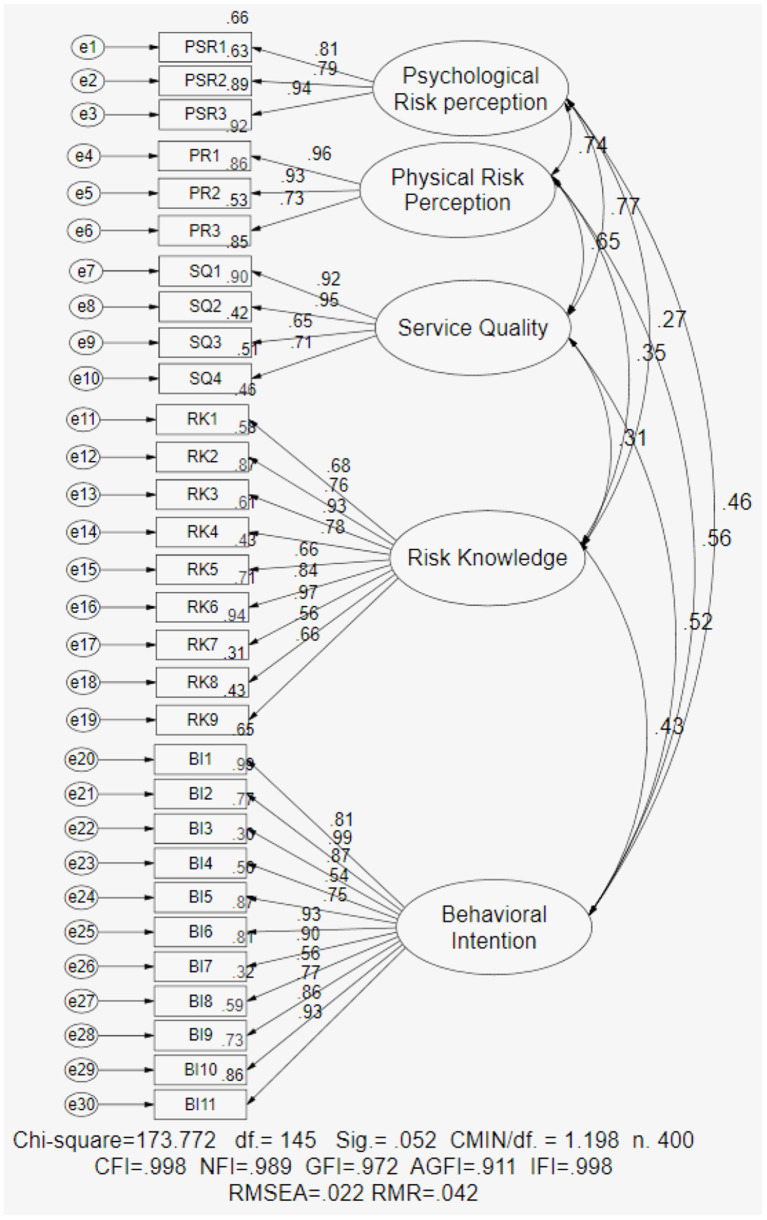
Confirmatory factor analysis (CFA) of the model of risk knowledge pathways to behavioral intention of air travel risk perception during COVID-19.

The analysis shows that the chi-square statistic was 173.772 (df = 145.00, Sig. = 0.052>0.05, CMIN/df. 1.198 <2.0) based on the results. The casual factors of stress resulting from the confirmatory factor analysis show that the model fits the data well, as evidenced by several fit statistics including a comparative fit index (CFI) of 0.998 > 0.90, a goodness-of-fit index (GFI) of 0.972 > 0.90, an adjusted goodness-of-fit index (AGFI) of 0.911 > 0.80, a root mean square error of approximation (RMSEA) of 0.022 <0.05, a root mean square residual (RMR) of 0.042 <0.08), an incremental fit index (IFI) of 0.998 > 0.90, and a normed fit index (NFI) of 0.989 > 0.05. In summary, the confirmatory factor analysis (CFA) of the risk knowledge pathways model to the behavioral intention of air travel risk perception during COVID-19 strongly suggests that each set of items represents a single underlying construct and provides evidence for discriminate validity or OK fit, as shown in Figure 5.

Table 3 contains the confirmatory factor analysis (CFA) of the model of risk knowledge pathways to the behavioral intention of air travel risk perception during COVID-19. The results indicate that the squared multiple correlation coefficients (R2) varied between 30.0 and 99.0%. The standardized factor loadings 0.55–0.99 were more than 0.50; the conclusion is compatible with the standardized factor loading concept proposed by Barclay et al. (1995). The average variance extracted (AVE) quantifies the variance captured by the indicators of measurement error; the results ranged from 0.593 to 0.768 more than 0.50 for convergent validity, which is an acceptable measurement (Hair et al., 2013). The discriminant validity is the measure of each construct differentiation, verified by the square root of AVE greater intercorrelation between the constructs (Byrne, 2010). Here, the construct's square root of AVE ranges from 0.770 to 0.876 (refer to Table 3) and is well above the intercorrelation range from 0.269 to 0.735 (refer to Table 4), verifying the discriminant validity of the study constructs. The composite reliability (CR) values for all constructs in the model ranging from 0.888 to 0.927 were more than 0.60, which is acceptable (Fornell and Larcker, 1981). The reliability from Cronbach's alpha coefficient ranged from 0.895 to 0.964, which is also more significant than 0.70 and therefore acceptable (Cronbach et al., 1990). All the resulting variables are acceptable values and strongly imply that each set of items represents a single underlying construct and provides evidence for discriminate validity or an OK fit confirmation; overall, the data indicate an excellent fit for the testing model.

**Table 3:** Analysis statistics of confirmatory factor analysis (CFA) of the model of risk knowledge pathways to behavioral intention of air travel risk perception during COVID-19.

**Variable**	**Factor loading**	**Error variances**	**t-value**	**R^2^**	**AVE**	**AVE**	**CR**	**Cronbach's alpha**
Psychological risk perception (PSR)					0.727	0.852	0.888	0.916
PSR1 (Parameters weight)	0.82	–	–	66.0%				
PSR2	0.79	0.04	25.978^**^	63.0%				
PSR3	0.94	0.06	20.506^**^	89.0%				
Physical risk perception (PR)					0.768	0.876	0.907	0.900
PR1 (Parameters weight)	0.96	–	–	92.0%				
PR2	0.93	0.02	37.226^**^	86.0%				
PR3	0.73	0.04	20.007^**^	53.0%				
Service quality (SQ)					0.672	0.819	0.889	0.895
SQ1 (Parameters weight)	0.92			90.0%				
SQ2	0.95	0.03	33.599^**^	44.0%				
SQ3	0.65	0.05	16.571^**^	85.0%				
SQ4	0.72	0.05	19.121^**^	51.0%				
Risk knowledge (RK)					0.593	0.770	0.927	0.923
RK1 (Parameters weight)	0.68	–	–	46.0%				
RK2	0.76	0.10	10.861^**^	58.0%				
RK3	0.93	0.08	15.176^**^	87.0%				
RK4	0.78	0.08	12.054^**^	61.0%				
RK5	0.66	0.04	22.466^**^	43.0%				
RK6	0.85	0.11	11.064^**^	71.0%				
RK7	0.97	0.09	15.397^**^	94.0%				
RK8	0.56	0.08	9.252^**^	31.0%				
RK9	0.66	0.08	12.828^**^	86.0%				
Behavioral intention (BI)					0.676	0.822	0.924	0.964
BI1 (Parameters weight)	0.81	–	–	65.0%				
BI2	0.99	0.05	26.319^**^	99.0%				
BI3	0.88	0.06	19.409^**^	77.0%				
BI4	0.55	0.06	12.942^**^	30.0%				
BI5	0.75	0.03	32.097^**^	56.0%				
BI6	0.93	0.05	22.798^**^	87.0%				
BI7	0.90	0.05	20.408^**^	81.0%				
BI8	0.57	0.06	12.945^**^	32.0%				
BI9	0.77	0.03	33.188^**^	73.0%				
BI10	0.86	0.02	45.270^**^	42.0%				
BI11	0.93	0.05	23.328^**^	59.0%				

** Significant at the 0.001 level.

**Table 4:** Analysis correlation variable of the model of risk knowledge pathways to behavioral intention of air travel risk perception during COVID-19.

**Variable**	**Behavioral intention (BI)**	**Service quality (SQ)**	**Risk knowledge (RK)**	**Physical risk perception (PR)**	**Psychological risk perception (PSR)**
Behavioral intention (BI)	1.000				
Service quality (SQ)	0.519^**^	1.000			
Risk knowledge (RK)	0.425^**^	0.310^**^	1.000		
Physical risk perception (PR)	0.559^**^	0.652^**^	0.348^**^	1.000	
Psychological risk perception (PSR)	0.463^**^	0.769^**^	0.269^**^	0.735^**^	1.000

** Significant at the 0.001 level.

### Analysis verifies the variable of the distribution of data

#### The distribution of measurements

Table 5 shows the descriptive data distribution analysis variables of the model of risk knowledge pathways to the behavioral intention of air travel risk perception during COVID-19. The analysis shows 30 observable variables including psychological risk perception (PSR), physical risk perception (PR), service quality (SQ), risk knowledge (RK), and behavioral intention (BI). The results show scores ranging from 3.00 to 4.00, a minimum of 1.00, and a maximum of 5.00. The standard deviations are all <1.5 (30% of mean); therefore, the data are not widely dispersed from the mean scores, which range from 3.56 to 4.24, and the standard deviation scores, which range from 0.64 to 1.04. The variance scores range from 0.41 to 0.95. For the measures of skewness and kurtosis, the results show negative skewness scores ranging from (−1.00) to (−0.02). Values for asymmetry and skewness between −2 and +2 are considered acceptable to prove normal univariate distribution (George and Mallery, 2010). The kurtosis scores range from (−1.15) to 0.70, and kurtosis is between (−7) and (+7) (Byrne, 2010). The coefficient of variation (CV), which is a statistical measure of the dispersion of data points in a data series around the mean scores, ranges from 15.82 to 27.43%, and the value range of 20–30% was acceptable (Griffiths, 1967). The results show that all variables with a normal distribution are considered acceptable when utilizing the model of risk knowledge pathways to the behavioral intention of air travel risk perception during COVID-19.

**Table 5:** Descriptive data distribution of the variable risk knowledge pathways to behavioral intention of air travel risk perception during COVID-19.

**Variable**	**Range**	**Min**	**Max**	**Mean**	**Std**.	**Variance**	**Skewness**	**Kurtosis**
Psychological risk perception (PSR)					
PSR1	3.00	2.00	5.00	4.22	0.86	0.75	−0.88	−0.03
PSR2	4.00	1.00	5.00	4.21	0.87	0.75	−0.99	0.48
PSR3	3.00	2.00	5.00	4.19	0.90	0.81	−0.87	−0.15
Physical risk perception (PR)							
PR1	3.00	2.00	5.00	3.95	0.84	0.70	−0.38	−0.53
PR2	3.00	2.00	5.00	4.01	0.77	0.60	−0.18	−0.88
PR3	3.00	2.00	5.00	3.95	0.81	0.66	−0.05	−1.15
Service quality (SQ)								
SQ1	3.00	2.00	5.00	4.05	0.64	0.41	−0.16	−0.16
SQ2	3.00	2.00	5.00	4.03	0.70	0.49	−0.57	0.70
SQ3	3.00	2.00	5.00	4.05	0.80	0.63	−0.60	0.00
SQ4	3.00	2.00	5.00	4.02	0.81	0.65	−0.35	−0.66
Risk knowledge (RK)								
RK1	3.00	2.00	5.00	3.56	0.98	0.95	−0.02	−1.00
RK2	3.00	2.00	5.00	3.70	0.93	0.86	−0.20	−0.82
RK3	3.00	2.00	5.00	3.80	0.90	0.81	−0.20	−0.83
RK4	3.00	2.00	5.00	3.79	0.85	0.73	−0.17	−0.70
RK5	3.00	2.00	5.00	3.64	0.97	0.95	−0.08	−1.00
RK6	3.00	2.00	5.00	3.75	0.92	0.84	−0.23	−0.79
RK7	3.00	2.00	5.00	3.82	0.89	0.80	−0.22	−0.80
RK8	3.00	2.00	5.00	3.81	0.89	0.79	−0.22	−0.79
RK9	3.00	2.00	5.00	3.79	0.96	0.93	−0.24	−0.97
Behavioral intention (BI)								
BI1	4.00	1.00	5.00	4.17	0.86	0.74	−0.88	0.24
BI2	4.00	1.00	5.00	4.12	0.89	0.79	−0.67	−0.37
BI3	4.00	1.00	5.00	4.24	0.89	0.80	−1.00	0.22
BI4	4.00	1.00	5.00	3.82	1.04	1.09	−0.54	−0.51
BI5	4.00	1.00	5.00	4.18	0.84	0.70	−0.84	0.22
BI6	4.00	1.00	5.00	4.13	0.86	0.74	−0.58	−0.51
BI7	3.00	2.00	5.00	4.24	0.86	0.74	−0.82	−0.32
BI8	4.00	1.00	5.00	3.92	0.97	0.95	−0.55	−0.36
BI9	4.00	1.00	5.00	4.19	0.85	0.72	−0.87	0.26
BI10	3.00	2.00	5.00	4.21	0.82	0.66	−0.78	−0.02
BI11	3.00	2.00	5.00	4.17	0.85	0.72	−0.64	−0.54

**Table 6:** Fit indices proposed for the model of risk knowledge pathways to behavioral intention of air travel risk perception during COVID-19.

**Index**	**Criteria**	**Result**	**References**	**Result**
Chi – Square = 221.229 df. = 189.0				
Sig.	>0.05	0.054	Bollen (1989); Sorbon (1996); Hair et al. (2010a,b,c)	Good fit
CMIN/df	<2.0	1.171	Hair et al. (2010a,b,c); Kelloway (2015)	Good fit
GFI	≥0.90	0.965	Browne and Cudeck (1993); Hair et al. (2010a,b,c)	Good fit
AGFI	≥0.80	0.914	Baumgartner and Hombur (1996); Zikmund (2003)	Good fit
NFI	≥0.90	0.986	Mueller (1996); Hu and Bentler (1999); Hair et al. (2010a,b,c)	Good fit
IFI	≥0.90	0.998	Bentler (1990); Hair et al. (2010a,b,c)	Good fit
CFI	≥0.90	0.998	Mueller (1996); Goffin (2007); Hair et al. (2010a,b,c)	Good fit
RMR	<0.08	0.040	Hu and Bentler (1999); Diamantopoulos and Siguaw (2000)	Good fit
RMSEA	<0.08	0.021	Hair et al. (2010a,b,c)	Good fit

#### The correlations of measurements

The results revealed this part to test the correlations measuring the determinant on the model of risk knowledge pathways to the behavioral intention of air travel risk perception during COVID-19. These variables were psychological risk perception (PSR), physical risk perception (PR), service quality (SQ), risk knowledge (RK), and behavioral intention (BI). The analysis results show that correlation coefficient scores ranging from 0.269 to 0.769 <0.80 are a good positive correlation and an acceptable measurement (Kline, 2011); the correlation is significant at the 0.001 level. Thus, the construct validity in this paper was ensured and did not present multicollinearity, and the data were revealed to explain the results of Table 3.

### Structural equation modeling

**Figure 6:**
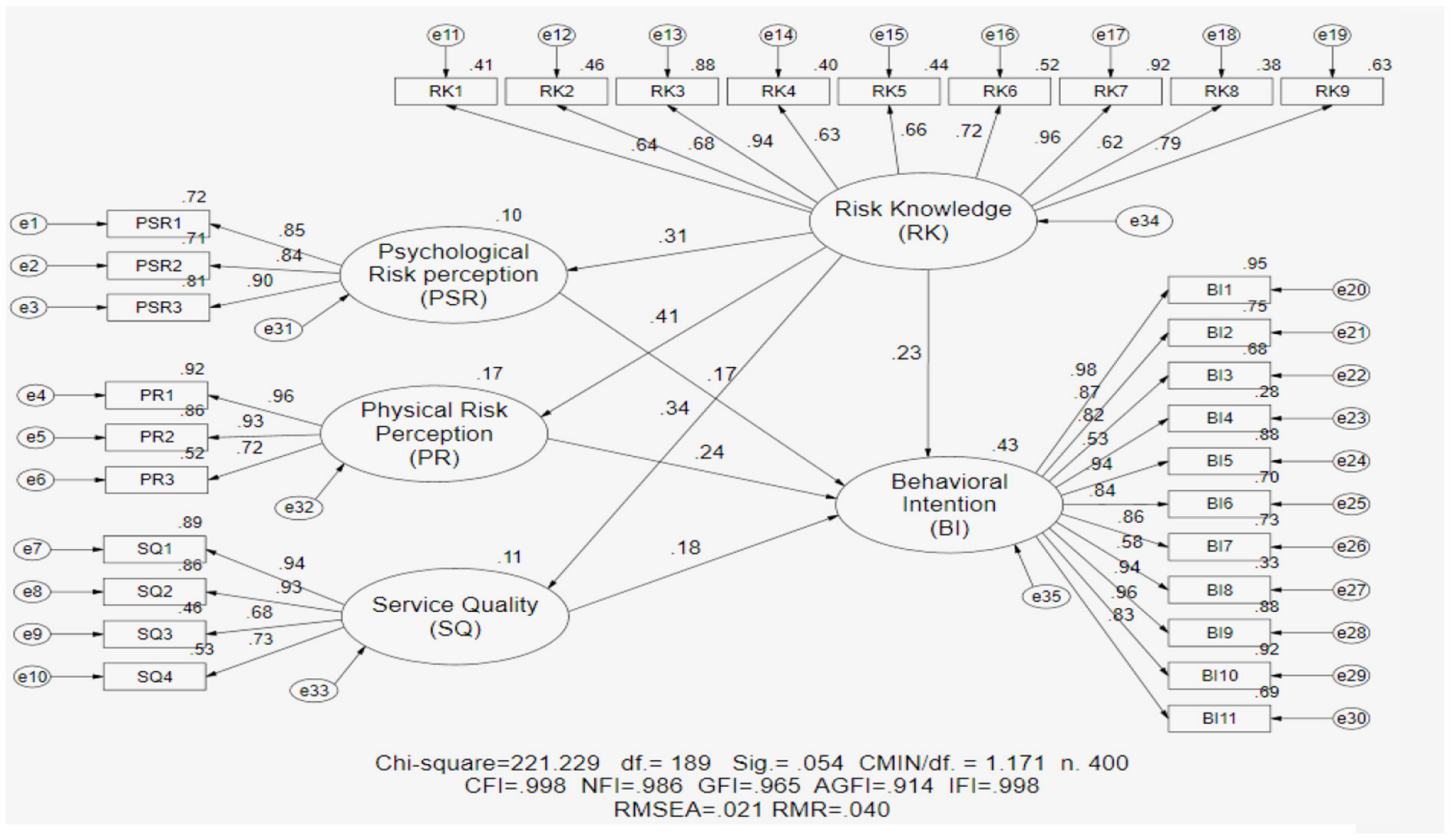
Structural equation modeling analysis for risk knowledge pathways to behavioral intention of air travel risk perception during COVID-19.

Structural equation modeling (SEM) has become one of the techniques of choice for researchers across disciplines. Therefore, this section investigates the model of risk knowledge pathways to the behavioral intention of risk perception during COVID-19 using the structural equation modeling (SEM) analysis technique to test the relationships among various constructs, as shown in Table 7 and Figure 6.

**Table 7:** Analysis statistics of the structural equation modeling analysis of risk knowledge pathways to behavioral intention of air travel risk perception during COVID-19.

**Variable**	**Path**	**Variable**	**λ**	**S.E**.	**t**.	**Sig**.	**R^2^**
Psychological risk perception (PSR)	< −−	Risk knowledge (RK)	0.31	0.06	6.071	0.000^*^	10.0%
Service quality (SQ)	<	Risk knowledge (RK)	0.34	0.05	6.828	0.000^*^	17.0%
Physical risk perception (PR)	< −−	Risk knowledge (RK)	0.41	0.07	8.217	0.000^*^	11.0%
Behavioral intention (BI)	< −−	Physical risk perception (PR)	0.24	0.06	4.420	0.000^*^	43.0%
Behavioral intention (BI)	< −−	Psychological risk perception (PSR)	0.17	0.07	2.726	0.006^*^	43.0%
Behavioral intention (BI)	< −−	Service quality (SQ)	0.18	0.09	2.788	0.005^*^	43.0%
Behavioral intention (BI)	< −−	Risk knowledge (RK)	0.23	0.06	5.360	0.000^*^	43.0%

* Significant at the 0.05 level.

The data revealed that the structural equation modeling (SEM) of risk knowledge pathways to the behavioral intention of air travel risk perception during the COVID-19 pandemic identifies five latent total variables including psychological risk perception (PSR), physical risk perception (PR), service quality (SQ), risk knowledge (RK), and behavioral intention (BI). The chi-square value is the traditional measure for evaluating overall model fit and assessing the magnitude of discrepancy between the sample and fitted covariance matrices (Hu and Bentler, 1999). A good model fit would provide an insignificant result at a 0.05 threshold (Barrett, 2007). The results found acceptable threshold levels and are consistent with the concepts of Bollen (1989), Sorbon (1996), and Hair et al. (2010a,b,c). The results show chi-square statistics of 221.229, df = 189.0, Sig. = 0.054>0.05, and CMIN/df. Furthermore, the results of the structural equation modeling analysis model risk knowledge pathways to behavioral intention of air travel risk perception during COVID-19 demonstrate a relatively reasonable fit of the seven indices of the model to the data based on several fit statistics. The details are as follows:

(1) The comparative fit index (CFI) analyzes the disparity between the data and the hypothesized model to determine how well the model fits the data. The results show a CFI of 0.998>0.90, which is consistent with the concept of Hair et al. (2010a,b,c), and a good comparative fit index should be more than 0.90. A CFI value of more than 0.95 is presently recognized as an indicative of a good fit (Hu and Bentler, 1999 and Goffin, 2007).
(2) The goodness-of-fit index (GFI) measures how well the predicted model fits the observed covariance matrix. The results show a GFI of 0.965>0.909, which is consistent with the concept of Mueller (1996) and Hair et al. (2010a,b,c). The goodness-of-fit index (GFI) is a measure of fit between the hypothesized model and the observed covariance matrix, and a good goodness-of-fit index should be more than 0.90 (Goffin, 2007).
(3) The adjusted goodness-of-fit index (AGFI), which is affected by the number of indicators of each latent variable, results showed an AGFI of 0.914 > 0.80 and between 0.80 and 0.90 and are considered a reasonable model consistent with the concept of Joreskog and Sorbom (1989). The GFI and AGFI range between 0 and 1, with a cutoff value of more than 0.80, exceeding the recommended level of 0.8, indicating acceptable fit (Zikmund, 2003) and generally indicating acceptable model fit (Hu and Bentler, 1995; Baumgartner and Hombur, 1996).
(4) The root mean square error of approximation (RMSEA) mean reduces the issues of sample size by analyzing the discrepancy between the hypothesized model, with optimally chosen parameter estimates, and the population covariance matrix (Hooper et al., 2008). The results showed an RMSEA of 0.021 <0.08, which suggests acceptable threshold levels consistent with the concept of Browne and Cudeck (1993) and Hair et al. (2006), and indicate acceptable model fit.
(5) The root mean square residual (RMR) is the square root of the discrepancy between the sample covariance matrix and the model covariance matrix. The results show an RMR of 0.040 <0.08, which is consistent with the concept of Hu and Bentler (1999). The standardized root mean square residual reduces difficulty in interpretation and ranges from 0 to 1, with a value of 0.05 or less being indicative of an acceptable model (Diamantopoulos and Siguaw, 2000).
(6) The normed fit index (NFI) analyzes the discrepancy between the chi-squared value of the hypothesized model and the chi-squared value of the null model (Bentler and Bonett, 1980). The results showed an NFI of 0.986>0.90, which is an acceptable threshold level consistent with the concept of Hair et al. (2010a,b,c). More recent suggestions indicate that the cutoff criteria should be an NFI with the values of more than 0.95 for this statistic to be a good fit (Hu and Bentler, 1999).
(7) Incremental fit indices (IFIs) are frequently utilized when evaluating the fit of structural equation models. They are predicated on a comparison of a target model's fit to a null model's fit. The results show an IFI of 0.998>0.90, an acceptable threshold level; values close to 1 indicate a perfect fit consistent with the concept of Hair et al. (2010a,b,c).

The results in the table show the structural equation modeling analysis model, a causal model of risk knowledge pathways to the behavioral intention of air travel risk perception during COVID-19, which strongly suggests that each set of items represents a single underlying construct and provides evidence for discriminate validity or an OK fit confirmation. Overall, the data indicate an excellent fit for the testing model.

### Hypothesis testing

This part studies the hypothesis testing analysis of the structural equation modeling analysis of risk knowledge pathways to the behavioral intention of air travel risk perception during COVID-19; the results were analyzed as follows (Table 8).

**Table 8:** Summary hypothesis testing analysis of risk knowledge pathways to behavioral intention of air travel risk perception during COVID-19.

	**Hypotheses**	**Effect**	**Result**	**Coefficients**	**R^2^**
H1	Risk knowledge significantly influences behavioral intention of air travel	Direct	Supported	0.23	43.0%
H2	Risk knowledge significantly influences perceived physical risk of air travel	Direct	Supported	0.41	11.0%
H3	Risk knowledge significantly influences perceived psychological risk of air travel	Direct	Supported	0.31	10.0%
H4	Risk knowledge significantly influences perceived service quality of air travel	Direct	Supported	0.34	17.0%
H5	Perceived physical risk significantly influences behavioral intention of air travel	Direct	Supported	0.24	43.0%
H6	Perceived psychological risk significantly influences behavioral intention of air travel	Direct	Supported	0.17	43.0%
H7	Perceived service quality significantly influences behavioral intention of air travel	Direct	Supported	0.18	43.0%
H8	Perceived physical risk significantly mediates risk knowledge and behavioral intention of air travel	Indirect	Supported	0.05	43.0%
H9	Perceived psychological risk significantly mediates risk knowledge and behavioral intention of air travel	Indirect	Supported	0.10	43.0%
H10	Perceived service quality significantly mediates risk knowledge and behavioral intention of air travel	Indirect	Supported	0.06	43.0%

^*^Significant at the 0.05 level.

The data revealed the hypothesis testing analysis for fit confirmation of the model structural equation modeling (SEM) analysis model of risk knowledge pathways to the behavioral intention of air travel risk perception during COVID-19. It consists of the following variables: psychological risk perception (PSR), physical risk perception (PR), service quality (SQ), risk knowledge (RK), and behavioral intention (BI), which summarize the path coefficients. The results reveal the following about the study's hypotheses.

H1. Risk knowledge significantly influences behavioral intention of air travel during the pandemic in Thailand.

The results show that risk knowledge significantly influences the behavioral intention of air travel during the pandemic in Thailand. They explain a regression weight estimate of standardized coefficients 0.23, a standard error of ~0.06, a *t*-value of 5.360, and a Sig. 0.000 <0.05. The risk knowledge influence to change the behavioral intention of the model was able to explain the influence to change at a rate of 43.0%; hence, H1 is supported. Risk knowledge significantly influenced the behavioral intention of air travel during the pandemic in Thailand, which was significant at or below the 0.05 level.

H2. Risk knowledge significantly influences the perceived physical risk of air travel during the pandemic in Thailand.

The results show that risk knowledge significantly influences the physical risk perception of air travel during the pandemic in Thailand. They explain a regression weight estimate of standardized coefficients of 0.41, a standard error of ~0.07, a *t*-value of 6.828, and a Sig. 0.000 <0.05. The risk knowledge influence to change the physical risk perception of the model was able to explain the influence to change at a rate of 11.0%; hence, H2 is supported. Risk knowledge significantly influenced the physical risk perception of air travel during the pandemic in Thailand, which was significant at or below the 0.05 level.

H3. Risk knowledge significantly influences the perceived psychological risk of air travel during the pandemic in Thailand.

The results show that risk knowledge significantly influences the perceived psychological risk of air travel during the pandemic in Thailand. They explain a regression weight estimate of standardized coefficients of 0.31, a standard error of ~0.06, a *t*-value of 6.071, and a Sig. 0.000 <0.05. The risk knowledge influence to change the perceived psychological risk of the model was able to explain the influence to change at a rate of 10.0%; hence, H3 is supported. Risk knowledge significantly influenced the perceived psychological risk of air travel during the pandemic in Thailand, which was significant at or below the 0.05 level.

H4. Risk knowledge significantly influences the perceived service quality of air travel during the pandemic in Thailand.

The results show that risk knowledge significantly influenced the perceived service quality of air travel during the pandemic in Thailand. They explain a regression weight estimate of standardized coefficients of 0.34, a standard error of ~0.05, a *t*-value of 6.828, and a Sig. 0.000 <0.05. The risk knowledge influence to change the perceived service quality of the model was able to explain the influence to change at a rate of 17.0%; hence, H4 is supported. Risk knowledge significantly influenced the perceived service quality of air travel during the pandemic in Thailand, which was significant at or below the 0.05 level.

H5. Perceived physical risk significantly influences the behavioral intention of air travel during the pandemic in Thailand.

The results show that perceived physical risk significantly influenced the behavioral intention of air travel during the pandemic in Thailand. They explain a regression weight estimate of standardized coefficients of 0.24, a standard error of ~0.06, a *t*-value of 4.420, and a Sig. 0.000 <0.05. The perceived physical risk influence to change the behavioral intention of the model was able to explain the influence to change at a rate of 43.0%; hence, H5 is supported. The perceived physical risk significantly influenced the behavioral intention of air travel during the pandemic in Thailand, which was significant at or below the 0.05 level.

H6. Perceived psychological risk significantly influences the behavioral intention of air travel during the pandemic in Thailand.

The results show that perceived psychological risk significantly influenced the behavioral intention of air travel during the pandemic in Thailand. They explain a regression weight estimate of standardized coefficients of 0.17, a standard error of ~0.07, a *t*-value 2.726, and Sig. 0.006 <0.05. The perceived psychological risk influence to change the behavioral intention of the model was able to explain the influence to change at a rate of 43.0%; hence, H6 is supported. The perceived psychological risk significantly influenced the behavioral intention of air travel during the pandemic in Thailand, which was significant at or below the 0.05 level.

H7. Perceived service quality significantly influences the behavioral intention of air travel during the pandemic in Thailand.

The results show that perceived service quality significantly influenced the behavioral intention of air travel during the pandemic in Thailand. They explain a regression weight estimate of standardized coefficients of 0.18, a standard error of ~0.09, a *t*-value of 2.788, and a Sig. 0.005 <0.05. The perceived service quality influence to change the behavioral intention of the model was able to explain the influence to change at a rate of 43.0%; hence, H7 is supported. The perceived service quality significantly influenced the behavioral intention of air travel during the pandemic in Thailand, which was significant at or below the 0.05 level.

H8. Perceived physical risk significantly mediates risk knowledge and behavioral intention of air travel during the pandemic in Thailand.

The results show that perceived physical risk significantly mediates risk knowledge and the behavioral intention of air travel during the pandemic in Thailand. They explain a regression weight estimate of risk knowledge to perceived physical risk coefficients of 0.41 and perceived physical risk to behavioral intention coefficients of 0.24. The risk knowledge to behavioral intention direct path coefficient was 0.31, and the indirect path coefficient was 0.10 (0.41 × 0.24). Hence, H8 is supported; the perceived physical risk significantly mediated risk knowledge and behavioral intention of air travel during the pandemic in Thailand, which was significant at or below the 0.05 level (Preacher et al., 2007).

H9. Perceived psychological risk significantly mediates risk knowledge and behavioral intention of air travel during the pandemic in Thailand.

The results show that perceived psychological risk significantly mediates risk knowledge and behavioral intention of air travel during the pandemic in Thailand. They explain a regression weight estimate of risk knowledge to perceived psychological risk coefficients of 0.31 and perceived psychological risk to behavioral intention coefficients of 0.17, and the risk knowledge to behavioral intention direct path coefficient was 0.31 along with the indirect path coefficient 0.052 (0.31 × 0.17). Hence, H9 is supported; the perceived psychological risk significantly mediated risk knowledge and behavioral intention of air travel during the pandemic in Thailand, which was significant at or below the 0.05 level.

H10. Perceived service quality significantly mediates risk knowledge and behavioral intention of air travel during the pandemic in Thailand.

The results show that perceived service quality significantly mediates risk knowledge and behavioral intention of air travel during the pandemic in Thailand. They explain a regression weight estimate of risk knowledge to perceived service quality of coefficients of 0.34 and perceived service quality to behavioral intention of coefficients of 0.18. The risk knowledge to behavioral intention direct path coefficient was 0.31 along with indirect path coefficients of 0.061 (0.34 × 0.18). Hence, H10 is supported; the perceived service quality significantly mediated risk knowledge and behavioral intention of air travel during the pandemic in Thailand, which was significant at or below the 0.05 level.

## Discussion and conclusion

### Discussion

The result of structural equation modeling (SEM) analysis models shows risk knowledge pathways to the behavioral intention of air travel of risk perception during COVID-19. The research found that the risk knowledge significantly influences the behavioral intention of air travel during the pandemic in Thailand, with a coefficient value of 0.23. The result is consistent with research, and Lepp and Gibson (2003) found that rich knowledge about travel, food, and health will operationally control perceived risk in the international travel circle. Additionally, the risk knowledge significantly influences the physical risk perception of air travel during the pandemic in Thailand, with a coefficient value of 0.41, and the results of the study based on the research by Wang and Xu (2020) found the significance of risk knowledge and public perception along with interest involvement and information saturation. In addition, the result found that the risk knowledge significantly influences the perceived psychological risk of air travel during the pandemic in Thailand, with a coefficient value of 0.31, and the risk knowledge significantly influences the perceived service quality of air travel during the pandemic in Thailand, with a coefficient value of 0.34. The result is consistent with the research by Stone and Grønhaug (1993) and Lou (2004) show that the travel industry documents, crises, and cultural and functional risks lift the constructs of sustainable traveler perception.

This study aimed to understand the sustainable air travel behavioral intentions in the context of the Thai aviation industry to determine how pandemics alter behavioral makeup. First, the empirical results mapped a significantly positive association between risk knowledge and behavioral intention to sustainable travel decisions. The prismatic mental foundation of the travelers folded under uncertainty, where the dissemination of knowledge drives behavioral development. Moreover, the social connectivity flow is at the stake, which asks the information source to be the center of gravity to survive and return to life on real grounds. The knowledge flow draws a line between favorable and unfavorable situations such as COVID-19, and knowledge excels as an optimistic way to travel for work or visit areas away from home *via* a physical journey (Temme et al., 2006; Daniali et al., 2015; Zhu and Deng, 2020).

Second, this work elucidates the significant contribution of risk knowledge to risk perception, with a more specific positive impact on physical, psychological, and service quality attributes to overcome the fear and arousal of integrative capacity in humans under challenging circumstances, which was also highlighted by Chai et al. (2011). Moreover, the sustainability of travelers' perceptions impacts the travel industry with regard to design knowledge fit. This situational knowledge demonstrates how to neutralize risk and promulgate countermeasures to deal with negative physical and psychological impacts. The aviation industry also reshapes its operations in accordance with preventive measures under the guidance of the WHO.

Third, according to Liou and Tzeng (2007) and Glanz et al. (2008), the path coefficients indicate a significant impact of psychological and physical risk perception on behavioral intention, and Chen (2008) supported a significant impact of service quality reported by the study. Here, the behavioral structures of the travelers explain the positivity of perception in terms of physical and psychological domains. Therefore, the reshaping human intention is possible in pandemics that are also historically reported to be accompanied by natural and economic disasters. People have returned to normal life behaviors in similar situations of life-threatening dangers such as SARS 2002 and the Ebola virus 2013–2016. Furthermore, positivity of perception under pandemic along with reliable quality service helps travelers to move toward their destination, supporting the claim of Shah et al. (2002).

Finally, empirical evidence showcases the reasoning that risk perception obtains the feasibility of travel willingness. This is the path highlighted by the work of Zeng et al. (2012), which explains the connectivity of knowledge to perceptual buildup contributing to behavioral development. Human psychology is proven critical in this study for curing the central point of recent discussion that is COVID-19. As long as humans hold strong norms to fight against this unforeseen enemy, every economic indicator will be back on track and contribute to sustainable development. Physical risk perception is also proven to connect risk knowledge and sustainable travel behavior, which is the ultimate damage of the disease. This might explain how people consider this fact; however, the neutralization of perception is key to safeguarding the behavioral path. International airlines reshaped their services to retain customers by adapting to the pandemic to stay operational. Here, many human intentions and work standards of the aviation industry in Thai circles play a connecting role in people's chain of safety to sustain traveling habits during pandemics. Ranking the mediating effect of the variables, physical risk had the highest significantly negative effect, followed by service quality with a positive significant effect and psychological risk with a minor negatively significant effect on the relationship of knowledge risk and travelers' behavioral intention. The overall testing of the theoretical base, knowledge, attitude, and behavior (KAB) theory was tested, and the study asserts that behavioral changes under the existence of knowledge are the foundation of the human behavioral makeup.

### Conclusion

This research is based on a deductive approach to an intact theoretical model with empirical evidence to examine the connectivity of risk knowledge reasoning and perception of risk in the behavioral intentions of travelers in Thailand. A survey is conducted by incorporating 399 respondents who travel through renowned airports using a variance-based SEM technique to generate empirical evidence to test the hypothesized relationship. The statistical analysis of this work suggests the predictive power of risk knowledge in sustainable travel behavior using aviation services. Second, the findings reveal a significant contribution of risk perception constructs, i.e., psychological, physical, and quality of service, to intention development during travel inside and outside Thailand. Finally, the empirical evidence promulgates the mediating connectivity of risk knowledge and behavioral intention *via* risk perception. Overall, the study explains that pneumonia and travel knowledge are the critical tools that will sustain cognitive makeup to facilitate an understanding of pandemics while continuing the life cycle with perceptual balance, resulting in people's willingness to travel under controlled working circumstances.

### Implications and limitations

The recent era of isolation modified positions on human safety and business survival. The current pandemic has shifted overall human conduct with the impacts on factors such as social connectivity, business demeanor, and travel. In the literature, various studies highlight the prevalence of pandemics along with causes and consequences in the academic and business research. Perceptual studies are limited in number that address the range of disasters that have been experienced in the recent past. Prior studies have analyzed the economic, civic, health, and educational impacts of pandemics across the globe. Considering the perception of the pandemic crisis, an impact mechanism of crisis knowledge on travelers' behavioral intention *via* the perception of risk was presented.

The academic side of the research has many implications. First, this study contributes to the field of service management by designing an impact mechanism of risk knowledge guiding service consumption behavior. Second, the study accumulated travel and pneumonia knowledge leading to behavioral intention, and previous pneumonia and tourism knowledge during COVID-19 are being investigated in the Chinese context. Third, the mediating factors are considered in this empirical work to highlight the reasoning path from risk knowledge to travel behavior *via* physical risk, psychological risk, and service quality. The empirical findings serve as a stepping stone by demonstrating the conceptual means of constructing knowledge in uncertain circumstances across the country. Finally, this research elaborates the underpinning behavioral components of intention, willingness, and recommendations for travel across the country given the prevalence of COVID-19.

The empirical findings of the study promulgated the personification of risk knowledge on behavioral makeup in the Thai aviation industry. Aviation industry management can add information to sustain travel and pandemic knowledge. This information channel will disseminate preventive measures of epidemics during travel. The perceptual development of travelers can be captured by mapping the lack of knowledge in the aviation industry by cultivating knowledge about uncertainty. This study provides critical insight for the aviation industry to redesign operation manuals in the consideration of external factors, and adaptive measures are required with the spread of the pandemic. A perceptual shift is what the Thai aviation industry needs to achieve sustainable local and international tourism, which could be made possible by channeling knowledge regarding viral disease, travel, physical, and psychological uncertainty, which demand a service quality shift and leading traveler behavior.

This paper makes a significant contribution to the sustainable travel behavior of passengers during prolonged uncertain traveling situations during COVID-19. Limitations include the study's findings being limited to six airports. Second, the limited sample of the paper could be extended in the future studies to obtain broader generalizing power to the population. Third, the paper's cross-sectional data can be shifted to longitudinal data for more in-depth analysis. Next, organizational cultural typologies are critical beliefs that can be included in the study model's moderation capacity to broaden future implications.

## Data availability statement

The original contributions presented in the study are included in the article/supplementary material, further inquiries can be directed to the corresponding author.

## Ethics statement

The studies involving human participants were reviewed and approved by Faculty of Management and Science Ethics Committee. The patients/participants provided their written informed consent to participate in this study.

## Author contributions

WN and KQ conceptualized the idea of the study design, wrote the original draft, and methodology. KQ and MT performed the review, editing, formal data analysis, validation and conducted the survey and worked on data, reviewing, and editing. All authors have read and agreed to the published version of the manuscript.

## Conflict of interest

The authors declare that the research was conducted in the absence of any commercial or financial relationships that could be construed as a potential conflict of interest.

## Publisher's note

All claims expressed in this article are solely those of the authors and do not necessarily represent those of their affiliated organizations, or those of the publisher, the editors and the reviewers. Any product that may be evaluated in this article, or claim that may be made by its manufacturer, is not guaranteed or endorsed by the publisher.

## References

[B1] Adrienne N. Budd L. Ison S. (2020). Grounded aircraft: An airfield operations perspective of the challenges of resuming flights post COVID. J. Air Transp. Manag. 89, 101921. 10.1016/j.jairtraman.2020.101921 32901184 PMC7470860

[B2] Aircraft Traffic Report (2020). Air Transport Information and Slot Coordination Division. Available online at: https://www.airportthai.co.th/wp-content/uploads/2020/08/Report-2019.pdf

[B3] Airport of Thailand PLC. (2020). AOT Air Traffic 8 months FY2020 - prelim (Oct 2019-Apr 2020).

[B4] Alrubaiee L. S. Alkaa'ida F. (2011). The mediating effect of patient satisfaction in the patients' perceptions of healthcare quality - patient trust relationship. Int. J. Market. Stud. 3, 103–127. Available online at: https://citeseerx.ist.psu.edu/viewdoc/download?doi=10.1.1.663.5764&rep=rep1&type=pdf31795011

[B5] Bae S. Y. Chang P. J. (2021). The effect of coronavirus disease-19 (COVID-19) risk perception on behavioural intention towards ‘untact'tourism in South Korea during the first wave of the pandemic (March 2020). Curr. Issues Tour. 24, 1017–1035. 10.1080/13683500.2020.1798895

[B6] Barclay D. Thompson R. Higgins C. (1995). The partial least squares (PLS) approach to causal modeling; Personal computer adoption and use as an Illustration. Technol. Stud. 2, 285–309. Retrieved from: https://www.researchgate.net/publication/242663837_The_Partial_Least_Squares_PLS_Approach_to_Causal_Modeling_Personal_Computer_Use_as_an_Illustration

[B7] Barrett P. (2007). Structural equation modelling: Adjudging model fit. Pers. Individ. Differ. 42, 815–824. 10.1016/j.paid.2006.09.018

[B8] Bateson J. E. G. Hoffman K. D. (2002). Fundamentos de marketing de servicios: conceptos, estrategias y casos. Económico administrativas. Thomson Publishers.

[B9] Baumgartner H. Hombur C. (1996). Applications of structural equation modeling in marketing and consumer research: a review. Int. J. Res. Mark. 13, 139–161. 10.1016/0167-8116(95)00038-0

[B10] Bellizzi M. G. Eboli L. Mazzulla G. (2019). Air transport service quality factors: a systematic literature review. Transp. Res. Procedia 45, 218–225. 10.1016/j.trpro.2020.03.010

[B11] Bentler P. M. (1990). Comparative fit indexes in structural models. Psychol. Bull. 107, 238–46. 10.1037/0033-2909.107.2.238 2320703

[B12] Bentler P. M. Bonett D. G. (1980). Significance tests and goodness of fit in the analysis of covariance structures. Psychol. Bull. 88, 588–606. 10.1037/0033-2909.88.3.588

[B13] Bollen K. A. (1989). Structural Equations with Latent Variables. New York: John Wiley and Sons.

[B14] Browne M. W. Cudeck R. (1993). “Alternative ways of assessing model fit,” in TestingStructural Equation Models, eds Bollen, K. A., and Long. S., (Newbury Park CA: Sage), 136–162.

[B15] Byrne B. M. (2010). Structural Equation Modeling with Amos: Basic Concepts, Applications, and Programming, 2nd Edn. New York, NY: Taylor and Francis Group.

[B16] Cameron T. A. James M. D. (1987). Estimating willingness to pay from survey data: an alternative pre-test-market evaluation procedure. J. Mark. Res. 24, 389–395. 10.1177/002224378702400406

[B17] Cater E. (2006). Ecotourism as a western construct. J. Ecoturism. 5, 23–39. 10.1080/14724040608668445

[B18] Chai S. S. Cao Y. M. Long C. F. (2011). A study on the factors affecting tourists' risk perception based on the multiple regression model. J Ocean Univ China 3, 55–62.

[B19] Chen C. F. (2008). Investigating structural relationships between service quality, perceived value, satisfaction, and behavioral intentions for air passengers: evidence from Taiwan. Transp. Res. A Policy Pract. 42, 709–717. 10.1016/j.tra.2008.01.007

[B20] Chen J. Zhu C. Luo, B. (2015). A combination of camshift algorithm and brisk feature point for real time moving target tracking. J. Chongqing Univ. Technol. Nat. Sci. 29, 112–119.

[B21] Chin W. W. Marcolin B. L. Newsted P. R. (2003). A partial least squares latent variable modeling approach for measuring interaction effects: Results from a Monte Carlo simulation study and an electronic-mail emotion/adoption study. Inf. Syst. Res. 14, 189–217. 10.1287/isre.14.2.189.16018

[B22] Cho E. Hu H. (2009). The effect of service quality on trust and commitment varying across generations. Int. J. Consum. Stud. 33, 468–476. 10.1111/j.1470-6431.2009.00777.x

[B23] Choi M. J. Kang M. Shin S. Y. Kim W. J. Jung J. Song J. Y. (2021). Comparison of antiviral effect for mild-to-moderate COVID-19 cases between lopinavir/ritonavir versus hydroxychloroquine: A nationwide propensity score-matched cohort study. Int. J. Infect. Dis. 102, 275–281. 10.1016/j.ijid.2020.10.062 33127507 PMC7590837

[B24] Conway III L. G. Woodard S. R. Zubrod A. (2020). Social Psychological Measurements of COVID-19: Coronavirus Perceived Threat, Government Response, Impacts, and Experiences Questionnaires. 10.31234/osf.io/z2x9a

[B25] Cronbach L. J. Hastorf A. H. Hilgard E. R. ansd Maccoby E. E. (1990). Robert R. Sears (1908-1989). Am. Psychol. 45, 663–664. 10.1037/h0091619

[B26] Cronin Jr J. J. Taylor S. A. (1992). Measuring service quality: a reexamination and extension. J. Mark. 56, 55–68. 10.1177/002224299205600304

[B27] Daniali S. S. Bakhtiari M. H. Nasirzadeh M. Aligol M. Doaei S. (2015). Knowledge, risk perception, and behavioral intention about hepatitis C, among university students. J. Educ. Health Promot. 4, 57–80. 10.4103/2277-9531.171807 27462635 PMC4946275

[B28] Diamantopoulos A. Siguaw J. A. (2000). Introduction to LISREL: A guide for the uninitiated. London: SAGE Publications, Inc.

[B29] Dowling G. R. Staelin R. (1994). A model of perceived risk and intended risk-handling activity. J. Consum. Res. 21, 119–134. 10.1086/209386

[B30] Durande-Moreau A. Usunier J. (1999). Time styles and the waiting experience: an exploratory study. J. Serv. Res. 2, 173–186. 10.1177/109467059922005

[B31] Fasanelli (2020). Social representations of Covid-19 in the framework of risk psychology. Papers Soc. Represent. 29, 8.1–8.36. Available online at: https://www.iris.unina.it/retrieve/handle/11588/832152/384904/PSR_COV_19-1182-2-10-20201231.pdf

[B32] Floyd M. F. Gibson H. Pennington-Gray L. Thapa B. (2004). The effect of risk perceptions on intentions to travel in the aftermath of September 11, 2001. J. Travel Tour. Mark. 15, 19–38. 10.1300/J073v15n02_02

[B33] Floyd M. F. Pennington-Gray L. (2004). Profiling risk perceptions of tourists. Ann. Tour. Res. 31, 1051–1054. 10.1016/j.annals.2004.03.011

[B34] Floyd R. Kirby E. (2001). Psychometric properties of measures of behavioral inhibition with preschool-age children: Implications for assessment of children at risk for ADHD. J. Attend. Disord. 5, 79–91. 10.1177/108705470100500202

[B35] Fornell C. Larcker D. F. (1981). Evaluating structural equation models with unobservable variables and measurement error. J. Mark. Res. 18, 39–50. 10.1177/002224378101800104

[B36] George D. Mallery P. (2010). SPSS for Windows Step by Step: A Simple Guide and Reference 17.0 Update, 10th Edn. Boston, MA: Pearson.

[B37] Glanz K. Rimer B. K. Viswanath K. (2008). Health Behavior And Health Education: Theory, Research, and Practice. San Francisco, CA: John Wiley and Sons.

[B38] Goffin R. D. (2007). Assessing the adequacy of structural equation model: Golden rules and editorial policy. Pers. Individ. Differ. 42, 831–839. 10.1016/j.paid.2006.09.019

[B39] Gong D. X. Du X. Y. (2019). Analysis on tourists' willingness in rural tourism and its influencing factors-Taking Huining county, Gansu province as an example. J. Res. Dev. Mark. 35, 1108–1112.

[B40] Griffiths J. C. (1967). Scientific Method in Analysis of Sediments. New York: McGraw - Hill Co. 109–73.

[B41] Guo F. Sullivan L. Wang H. Wang X. Li Z. Zhao Y. (2015). Enhancing phytolith carbon sequestration in rice ecosystems. Sci. Bull. 60, 591–597. 10.1007/s11434-015-0729-8

[B42] Hair J. Ringle C. Sarstedt M. (2013). Partial least squares structural equation modeling: rigorous applications, better results and higher acceptance. Long Range Plann. 46, 1–12. 10.1016/j.lrp.2013.08.016

[B43] Hair J. F. Anderson R. E. Tatham R. L. Black W. C. (2010b). Multivariate Data Analysis: A Global Perspective (7th Edition). Upper Saddle River, New Jersey: Pearson Prentice Hall.

[B44] Hair J. F. Black W. C. Babin B. J. Anderson R. E. (2010c). Multivariate data analysis (7ed.). Upper Saddle River, NJ, USA: Prentice-Hall, Inc.

[B45] Hair J. F. Black W. C. Babin B. J. Anderson R. E. Tatham R.L. (2006). Multivariate Data Analysis, 6^th^ Edn. Hoboken, NJ: Pearson Prentice Hall.

[B46] Hair J. F. J. Anderson R. E. Tatham R. L. Black W. C. (2010a). Multivariate Data Analysis (Sixth ed.). Upper Saddle River, New Jersey: Prentice Hall.

[B47] Han J. Y. (2005). The Relationships of Perceived Risk to Personal Factors, Knowledge of Destination, and Travel Purchase Decisions in International Leisure Travel. Blacksburg, VA: Virginia Polytechnic Institute and State University.

[B48] Hanemann W. M. (1991). Willingness to pay and willingness to accept: How much can they differ? *Am*. Econ. Rev. 81, 635–647. Available online at: http://www.jstor.org/stable/2006525

[B49] Harrison-Walker L. J. (2001). The measurement of word-of-mouth communication and investigation of service quality and customer commitment as potential antecedents. J. Serv. Res. 4, 60–75. 10.1177/109467050141006

[B50] Hooper D. Coughlan J. Mullen M. R. (2008). Structural equation modeling: Guidelines for determining model fit. J. Bus. Res. Methods 6, 53–60. Available online at: https://academic-publishing.org/index.php/ejbrm/article/view/1224/118726547545

[B51] Hu L. Bentler P. M. (1995). “Evaluating model fit,” in Structural Equation Modeling: Concepts, Issues and Applications, eds Hoyle, R. H., (Newbury Park, CA: Sage).

[B52] Hu L. Bentler P. M. (1999). Cutoff criteria for fit indexes in covariance structure analysis: Conventional criteria versus new alternatives. Struct. Equ. Model. 6, 1–55. 10.1080/10705519909540118

[B53] Hua H. Y. Liu S. M. Li W. (2010). A study on the correlations between the reasons for tourist churn in scenic areas and tourists' travel intention after serious natural disasters: a case study of Sichuan tourism industry after Wenchuan earthquake. Hum. Soc. Sci. J. Hainan Univ. 4, 80–86. 10.15886/j.cnki.hnus.2010.04.016

[B54] Hutchinson J. Lai F. Wang Y. (2009). Understanding the relationships of quality, value, equity, satisfaction, and behavioral intentions among golf travelers. Tour. Manag. 30, 298–308. 10.1016/j.tourman.2008.07.010

[B55] IATA International Air Transport Association. (2019). Annual Review 2019. 75th Annual General Meeting, Seoul, June 2019. IATA International Air Transport Association.Available online at: https://www.iata.org

[B56] IATA International Air Transport Association. (2020). Annual Review 2020. 76th Annual General Meeting, Amsterdam, November 2020. IATA International Air Transport Association.Available online at: https://www.iata.org

[B57] Jia A. S. (2018). Study on influencing factors of rural residents' tourism intention based on Probit model: taking Zhengzhou as an example. J. Shangqiu Voc. Tech. Coll 17, 47–51.

[B58] Joffe H. (2003). Risk: From perception to social representation. Br. J. Soc. Psychol. 42, 55–73. 10.1348/014466603763276126 12713756

[B59] Johnson M. S. Sivadas E. Garbarino E. (2008). Customer satisfaction, perceived risk and affective commitment: an investigation of directions of influence. J. Serv. Mark. 23, 45–60. 10.1108/08876040810889120

[B60] Joreskog K. G. Sorbom D. (1989). LISREL 7: A Guide to the Program and Applications. Chicago, IL: SPSS, Inc.

[B61] Kelloway E. K. (2015). Using Mplus for Structural Equation Modeling; A Researcher's Guide. CA: Sage Publications.

[B62] Kline R. B. (2011). Principles and Practice of Structural Equation Modeling. edition 3rd Ed. New York, NY: The Guilford Press.

[B63] Lamb T. L. Winter S. R. Rice S. Ruskin K. J. Vaughn A. (2020). Factors that predict passengers willingness to fly during and after the COVID-19 pandemic. J. Air Trans. Manag. 89, 101897. 10.1016/j.jairtraman.2020.101897 32837029 PMC7434314

[B64] Lepp A. Gibson H. (2003). Tourist roles, perceived risk and international tourism. Ann. Tour. Res. 30, 606–624. 10.1016/S0160-7383(03)00024-023744805

[B65] Li F. (2008). A study on the factors about tourism risk sense based on Logit model: a case study of earthquake in Sichuan on May12. Tour. Forum 1, 341–346.

[B66] Liou D. Smith M. M. (2007). Macroeconomic variables and financial distress. J. Account. Bus. Manag. 14. Available online at: http://journal.stie-mce.ac.id/index.php/jabminternational/article/view/292 (accessed August 5, 2022).

[B67] Liou J. J. Tzeng G. H. (2007). A non-additive model for evaluating airline service quality. J. Air Trans. Manag. 13, 131–138. 10.1016/j.jairtraman.2006.12.002

[B68] Liu C. (2019). The power of knowledge: reflections on the influencing factors of public risk perception-An exploratory analysis based on a popular science intervention experiment and survey. Shandong Soc. Sci. 11, 96–109.

[B69] Lou S. D. (2004). Tourism risk and prevention. Bus. Econ. 119–120, 127.

[B70] MacInnis D. J. Moorman C. Jaworski B. J. (1991). Enhancing and measuring consumers' motivation, opportunity, and ability to process brand information from ads. J. Mark. 32–53. 10.2307/1251955

[B71] Miller C. J. Smith S. N. Pugatch M. (2020). Experimental and quasi-experimental designs in implementation research. Psychiatr. Res. 283, 112452. 10.1016/j.psychres.2019.06.027 31255320 PMC6923620

[B72] Mitchell V. W. Vassos V. (1998). Perceived risk and risk reduction in holiday purchases: a cross-cultural and gender analysis. J. Euromark. 6, 47–79. 10.1300/J037v06n03_03

[B73] Mitra K. Reiss M. C. Capella L. M. (1999). An examination of perceived risk, information search and behavioral intentions in search, experience and credence services. J. Serv. Mark. 9, 110–125. 10.1108/08876049910273763

[B74] Mo F. Choi B. C. K. Clottey C. LeBrun B. Robbins G. (2002). Charecteristics and risk factors for accident injury in canada from 1986 to 19996: An analysis of the canadian accident injury reporting and evaluation (caire) database. Inj. Cont. Safe. Promot. 9, 73–81. 10.1076/icsp.9.2.73.8701 12461833

[B75] Molinari L. K. Abratt R. Dion P. (2008). Satisfaction, quality and value and effects on repurchase and positive word-of-mouth behavioral intentions in a B2B services context. J. Serv. Mark. 22, 363–373. 10.1108/08876040810889139

[B76] Moutinho L. (1987). Consumer behaviour in tourism. Eur. J. Market. 21, 5–44. 10.1108/EUM0000000004718

[B77] Mowen J. Minor M. (1988). Consumer Behavior, 5th ed. Englewood Cliffs, NJ, USA: Prentice Hall, 176.

[B78] Mueller R. O. (1996). “Confirmatory factor analysis,” in Basic Principles of Structural Equation Modeling: An Introduction to LISREL and EQS. New York: Springer-Verlag, 62–128.

[B79] Nania R. (2020). Blacks, Hispanics Hit Harder by the Coronavirus, Early US Data Show. Washington, DC: AARP.

[B80] Park J. W. Ryu Y. K. (2019). Investigating the effects of airport servicescape on airport users' behavioral intentions: a case study of Incheon international airport terminal 2 (T2). Sustainability 11, 4171. 10.3390/su11154171

[B81] Podsakoff P. M. MacKenzie S. B. Podsakoff N. P. (2012). Sources of method bias in social science research and recommendations on how to control it. Ann. Rev. Psychol. 63, 539–569. 10.1146/annurev-psych-120710-100452 21838546

[B82] Preacher K. J. Rucker D. D. Hayes A. F. (2007). Addressing moderated mediation hypotheses: theory, methods, and prescriptions. Multivar. Behav. Res. 42, 185–227. 10.1080/00273170701341316 26821081

[B83] Rice S. Winter S. R. Capps J. Trombley J. Robbins J. Milner M.. (2020). Creation of two valid scales: willingness to fly in an aircraft and willingness to pilot an aircraft. Int. J. Aviat. Aeronaut. Aerosp. 7, 5. 10.15394/ijaaa.2020.1440

[B84] Roehl W. S. Fesenmaier D. R. (1992). Risk perceptions and pleasure travel: an exploratory analysis. J. Travel Res. 30, 17–26. 10.1177/004728759203000403

[B85] Rosenstock I. M. (1974). Historical origins of the health belief model. Health Educ. Monogr. 2, 328–335. 10.1177/109019817400200403 299611

[B86] Ryu K. Lee H. R. Kim W. G. (2012). The influence of the quality of the physical environment, food, and service on restaurant image, customer perceived value, customer satisfaction, and behavioral intentions. Int. J. Contemp. Hosp. Manag. 20, 80–98. 10.1108/09596111211206141

[B87] Shah F. T. Syed Z. Imam A. Raza A. (2020). The impact of airline service quality on passengers' behavioral intentions using passenger satisfaction as a mediator. J. Air Trans. Manag. 85, 101815. 10.1016/j.jairtraman.2020.101815

[B88] Shah J. Y. Friedman R. Kruglanski A. (2002). forgetting all else: On the antecedents and consequences of goal shielding. J. Pers. Soc. Psychol. 83, 1261–1280. 10.1037/0022-3514.83.6.1261 12500810

[B89] Shepardson D. Reese C. Ellis A. (2020). American Airlines, Delta, United to Require Facial Coverings on US Flights. New York Times. Available online at: https://www.nytimes.com/reuters/2020/04/30/us/30reuters-health-coronavirus-usa-airlines.html (accessed July 19, 2021).

[B90] Singh T. Smith D. (2005). Direct-to-consumer prescription drug advertising: a study of consumer attitudes and behavioral intentions. J. Consum. Mark. 15, 45–70. 10.1108/07363760510631101

[B91] Sjöberg L. (1998). Worry and risk perception. Risk Anal. 18, 85–93. 10.1111/j.1539-6924.1998.tb00918.x 9523446

[B92] Song K. H. Choi S. (2021). A study on the perception change of passengers on sustainable air transport following COVID-19 progress. Sustainability 13, 8056. 10.3390/su13148056

[B93] Sorbon D. (1996). LISREL8: User's Reference Guide. Chicago, IL: Scientific Software International. p. 17. Available online at: www.sscientral.com

[B94] Spitzmuller C. Krishnamoorti R. Flin R. Datta A. (2020). The Energy Workforce and COVID-19: Data-Driven Policy Recommendations. Houston, TX, USA: University of Houston.

[B95] Stone R. N. Grønhaug K. (1993). Perceived risk: further considerations for the marketing discipline. Eur. J. Mark. 27, 39–50. 10.1108/03090569310026637

[B96] Taylor S. Landry C. A. Paluszek M. M. Fergus T. A. McKay D. Asmundson G. J. (2020). COVID stress syndrome: Concept, structure, and correlates. Depress. Anxiety. 37, 706-714. 10.1002/da.23071 32627255 PMC7362150

[B97] Temme D. Kreis H. Hildebrandt L (2006). PLS Path Modeling: A Software Review. SFB 649 Discussion Paper, No. 2006084. p. 1–20. Humboldt University of Berlin, Berlin, Germany.

[B98] Teo T. S. H. Srivastava S. C. Jiang L. (2009). Trust and electronic government success: an empirical study. J. Manag. Inf. Syst. 25, 103–137. 10.2753/MIS0742-1222250303

[B99] Unnikrishnan C. S. (2020). “A new gravitational paradigm for relativity and dynamics, and its philosophical scope,” in Journal of Physics: Conference Series, Vol. 1466 (IOP Publishing).

[B100] Van Fan Y. Jiang P. Hemzal M. Kleme,š J. J. (2021). An update of COVID-19 influence on waste management. Sci. Total Environ. 754, 142014. 10.1016/j.scitotenv.2020.142014 32920389 PMC7448788

[B101] Wang B. Titov I. Lapata M. (2019). “Learning semantic parsers from denotations with latent structured alignments and abstract programs,” in Proceedings of the 2019 Conference on Empirical Methods in Natural Language Processing and the 9th International Joint Conference on Natural Language Processing (Hong Kong: Association for Computational Linguistics), 3774–3785. 10.18653/v1/D19-1391

[B102] Wang G. Xu Y. Q. (2020). The influence factor of public perceived risk: a two-dimensional examination from interest and information——based on the empirical analysis of L city. J. Northeast. Univ. 22, 73. 10.15936/j.cnki.1008-3758.2020.01.010

[B103] Whitely A. Philip V. Jasper C. Schlangestine M. Truong A. (2020). How Coronavirus Will Forever Change Airlines and the Way We Fly. Hyperdrlve: Bloomberg.

[B104] Xu F. Li S. S. Niu W. X. Lin X. J. (2019). How to manage tourist destination risk effectively? Evidence from southern Xinjiang. Nankai Bus. Rev. 1, 66–75.

[B105] Yamane T. (1967). Statistics: An Introductory Analysis, 2nd Edn. New York, NY: Harper and Row. p. 2-5.

[B106] Yingzhi G. U. O. Yun C. H. E. N. Jianfeng H. U. A. N. G. Yong S. U. (2015). Travel Intentions of Chinese Residents to Japan Based on A Multidimensional Interactive Decision Tree Model. Tour. Tribune 30, 35–45.

[B107] Yu Y. Song C. Zhang Q. Dimaggio P. A. Garcia B. A. York A.. (2012). Histone H3 lysine 56 methylation regulates DNA replication through its interaction with PCNA. Mol. Cell. 46, 7–17. 10.1016/j.molcel.2012.01.019 22387026 PMC3327800

[B108] Zeithaml V. (1988). Consumer perceptions of price, quality and value: A means-end model and synthesis of evidence. J. Market. 52, 2–22. 10.1177/002224298805200302

[B109] Zeithaml V. A. Berry L. L. Parasuraman A. (1996). The behavioral consequences of service quality. J. Mark. 60, 31–46. 10.1177/002224299606000203

[B110] Zeng W. -C. Zhang Z. Gao H. Jia L. -R. Chen W. -Y. (2012). Characterization of antioxidant polysaccharides from auricularia auricular using microwave-assisted extraction. Carbohydrate Polym. 89, 694—700. 10.1016/j.carbpol.2012.03.078 24750775

[B111] Zeng Z. Wang X. Wang Z. Guo R. Feng R. (2017). Empirical research of the relationship between related knowledge, attitude and behavior of hypertension patients based on the structural equation model. Zhong Nan Da Xue Xue Bao Yi Xue Ban=J. Cent. South Univ. Med. Sci. 42, 195–201. 28255123 10.11817/j.issn.1672-7347.2017.02.013

[B112] Zhang J. Y. Guo X. R. Wu X. W. (2018). KAP Investigation and influential factor study of medication risk among residents. Chin. Phar 29, 1445–1448.

[B113] Zhang P. C. Chi X. L. Wu M. X. (2012). A study of characteristics and relationship among sexual health knowledge, sexual attitude and sex-related behavior in Chinese college students. Chin. J. Clin. Psychol 20, 849–853.

[B114] Zhang T. H. Cheng Y. J. (2008). A review of consumer perceived risk theory. Mark. Herald 4, 40–44.

[B115] Zhao Y. L. Mao D. W. Zhong L. L. (2016). The influence of service quality in ethnic villiages tourism on tourists' behavioral intention. J. Sichuan Norm. Univ. Soc. Sci 4, 80–89.

[B116] Zhao Y. N. (2012). Research on the influence of information factor on risk attitude of college students. Educ. Res. Mon. 11, 76–79.22783683

[B117] Zhu G'. Wang L. -L. Zhang X. -M. Shi W. X. Furuta K. (1988). Problems on management of specific pathogen free (SPF) chickens maintained in China and their performance. Jpn. Poult. Sci. 25, 241–248.

[B118] Zhu H. Deng F. (2020). How to influence rural tourism intention by risk knowledge during COVID-19 containment in China: Mediating role of risk perception and attitude. Int. J. Environ. Res. Public Health 17, 3514. 10.3390/ijerph17103514 32443430 PMC7277590

[B119] Zikmund W. G. (2003). Business Research Methods, 7th ed. Mason, OH: Thomson: South-Western.

